# Laxative use and incident falls, fractures and change in bone mineral density in postmenopausal women: results from the Women’s Health Initiative

**DOI:** 10.1186/1471-2318-13-38

**Published:** 2013-05-01

**Authors:** Bernhard Haring, Mary Pettinger, Jennifer W Bea, Jean Wactawski-Wende, Ryan M Carnahan, Judith K Ockene, Moritz Wyler von Ballmoos, Robert B Wallace, Sylvia Wassertheil-Smoller

**Affiliations:** 1 Department of Internal Medicine I, Comprehensive Heart Failure Center, University of Würzburg, Oberdürrbacher Strasse 6, Würzburg, 97080, Germany; 2 Fred Hutchinson Cancer Research Center, Seattle, Washington, USA; 3 Arizona Cancer Center, University of Arizona, Tucson, Arizona, USA; 4 Department of Social and Preventive Medicine, University at Buffalo, SUNY School of Public Health and Health Professions, Buffalo, NY, USA; 5 Department of Epidemiology, University of Iowa College of Public Health, Iowa City, Iowa, USA; 6 Division of Preventive and Behavioral Medicine, University of Massachusetts Medical School, Worcester, Massachusetts, USA; 7 Department of Surgery & Division of Cardiothoracic Surgery, Froedtert Memorial Hospital & Medical College of Wisconsin, Milwaukee, WI, USA; 8 Department of Epidemiology and Population Health, Albert Einstein College of Medicine, Bronx, New York, USA

**Keywords:** Laxative use, Falls, Fractures, Bone mineral density, Aging

## Abstract

**Background:**

Laxatives are among the most widely used over-the-counter medications in the United States but studies examining their potential hazardous side effects are sparse. Associations between laxative use and risk for fractures and change in bone mineral density [BMD] have not previously been investigated.

**Methods:**

This prospective analysis included 161,808 postmenopausal women (8907 users and 151,497 nonusers of laxatives) enrolled in the WHI Observational Study and Clinical Trials. Women were recruited from October 1, 1993, to December 31, 1998, at 40 clinical centers in the United States and were eligible if they were 50 to 79 years old and were postmenopausal at the time of enrollment. Medication inventories were obtained during in-person interviews at baseline and at the 3-year follow-up visit on everyone. Data on self-reported falls (≥2), fractures (hip and total fractures) were used. BMD was determined at baseline and year 3 at 3 of the 40 clinical centers of the WHI.

**Results:**

Age-adjusted rates of hip fractures and total fractures, but not for falls were similar between laxative users and non-users regardless of duration of laxative use. The multivariate-adjusted hazard ratios for any laxative use were 1.06 (95% confidence interval [CI], 1.03-1.10) for falls, 1.02 (95% CI, 0.85-1.22) for hip fractures and 1.01 (95% CI, 0.96-1.07) for total fractures. The BMD levels did not statistically differ between laxative users and nonusers at any skeletal site after 3-years intake.

**Conclusion:**

These findings support a modest association between laxative use and increase in the risk of falls but not for fractures. Its use did not decrease bone mineral density levels in postmenopausal women. Maintaining physical functioning, and providing adequate treatment of comorbidities that predispose individuals for falls should be considered as first measures to avoid potential negative consequences associated with laxative use.

## Background

Laxatives belong to one of the most frequently sold over-the-counter pharmacy sales categories in the United States [1]. In 2011, consumers spent approximately $875 million on laxative drug products [1]. This easy availability can facilitate use without indication, long-term use, inappropriate dosing and even abuse of these medicines [2-4]. Electrolyte and fluid imbalances have been reported as potential negative consequences and side-effects of laxative use [2-7]. These medical conditions are known causes of muscle weakness, lassitude, cardiac arrhythmias, and osteoporosis that may increase the risk for falls and fractures [8-14]. In fact, use of a wide range of prescription drugs such as loop diuretics or antiepileptics have already been shown to be associated with falls, fractures and even changes in bone mineral density as these drugs may alter fluid and electrolyte metabolism (e.g. hyponatremia, hypocalemia) and even vitamin D absorption [10,15-19].

To this point, prior epidemiological studies suggest that laxative use increases the risk for falls [2,20-29]. However, these studies were limited by small numbers of falls, lack of data on important potential confounders and short follow-up period [20,22,27,29]. Additionally, although electrolyte and fluid imbalances, vitamin malabsorption as well as a history of falls are important risk factors for changes in bone mineral density and fractures [9,10,30-39], the effect of laxative use on these bone outcomes remains to be determined.

Based on current literature laxative users may be characterized by several different groups [4,40,41]. The first and by far largest group is made up of individuals who are generally middle aged or older who begin using laxatives when constipated. The second group consists of individuals suffering from an eating disorder such as anorexia or bulimia nervosa. Third, individuals engaged in certain types of sports training may be inclined to be users. The fourth group includes surreptitious laxative abusers who use the drugs to cause factitious diarrhea. As constipation occurs more frequently in the female population and it becomes more prevalent with increasing age [42-45], the role of laxative use in postmenopausal women is of particular interest.

Our aim is to evaluate a possible association of laxative intake with the incidence of falls, total fractures, hip fractures and change in bone mineral density in postmenopausal women. Such information may provide insight into the safety profile and potential hazards of a widely used medication class and thereby be of high public health relevance.

## Methods

### Study population

The study population consisted of 161,808 women enrolled in the Women’s Health Initiative (WHI) Observational Study (OS) (n=93,676) and Clinical Trials (CTs) (n=68,132). Details of the study population have been described elsewhere [46-50]. For this analysis, 160,404 women with no prior hip fracture (OS: n=92,789/CT: n=67,615) were included. Women were recruited from October 1, 1993, to December 31, 1998, at 40 clinical centers in the United States and were eligible if they were 50 to 79 years old and were postmenopausal at the time of enrollment. In addition to age and menopausal status, eligibility criteria for the clinical trial (CT) and observational study (OS) included ability and willingness to provide written informed consent and an agreement to reside in the area for at least 3 years after enrollment [51], [46]. Participants were subsequently followed up through March 31, 2005, for an average of 7.8 years (OS: 7.6 years /CT: 8 years). Among 160,404 women, 7421 (4.6%) were lost to follow-up (OS: n=4451/ CT: n=2970), whereas 9709 (6.1%) deceased (OS: n=6146 /CT: n= 3563). OS participants were scheduled for two clinic visits at baseline and year 3 of follow-up compared to CT participants who were scheduled for clinic visits at years 1, 3, 6 and 9. To update selected exposures and ascertain medical outcomes participants in the OS were mailed annual questionnaires whereas women in the CTs were sent semiannual forms to ensure the timely update of medical histories [46]. All protocols were approved by institutional review boards at participating institutions.

### Exposure assessment

Current medication use was assessed by asking the participants to present all containers for medications taken for 2 weeks prior to clinic visit [46]. Clinic interviewers then entered each medication into the WHI database, which assigned drug codes using Medispan software (First DataBank, Inc., San Bruno, California). For this analysis, we used baseline information on current laxative use including label product name, label generic name, dosage form, strength and duration (WHI Form 44: Current Medications). Duration of use was examined in three categories (<1 year, 1 to 3 years, or >3years). Since the medication class of laxatives is a heterogeneous entity with various different kinds of pharmacologically active substances and with many commercially available laxative products consisting of two or more active pharmacological compounds, we investigated the effect of ‘any (compound containing) laxative’ on our outcomes of interest. We additionally stratified laxatives by the underlying containing substances into five categories [‘oral stimulant containing’, ‘fiber containing’, ‘osmotic containing’, ‘stool softener containing’ and ‘lubricant containing’ laxative] to potentially gain information on the distribution of these categories being used and the effects on our outcomes of interest. However, as we are lacking information on the exact composition and pharmacological impact of each compound of the various commercially available mixture products, for analyses these categories were not mutually exclusive.

### Outcome assessment and other covariates

As primary outcome measures, we assessed the incidence of falls and fractures (total and hip fractures) and change in bone mineral density. Incident falls and fractures were ascertained prospectively using annual (OS) or semiannual (CT) self-report questionnaires. Fall history was obtained by asking about the number of times the participant fell or landed on the ground (excluding falls owing to sports) in the interval since the last medical history update (WHI Form 33: Medical History). A participant was identified as having a history of falls if she reported 2 or more falls per year as previously defined in epidemiologic studies [15,16,52,53]. In WHI CTs, all fracture outcomes were verified by central review of radiology reports whereas in the WHI OS, only hip fractures were centrally adjudicated for all women.

Bone mineral density (BMD) at the total hip, posterior-anterior spine, and total body was measured at baseline at 3 of the 40 clinical centers of the WHI (Pittsburgh, Pennsylvania; Birmingham, Alabama; and Phoenix and Tucson, Arizona) among 10,833 women with dual-energy x-ray absorptiometry using a Hologic QDR densitometer (Hologic Inc, Waltham, Massachusetts). We determined change in BMD by laxative use at these sites from baseline to year 3. Information on all covariates was assessed via self-report, clinic interview or by physical measure at baseline [54-56].

### Statistical analysis

The characteristics of women using laxatives at baseline were compared with nonusers [Table 1]. Differences between laxative users and nonusers were compared using χ^2^ statistics for categorical variables and *t-*test or ANOVA, as appropriate, for continuous variables. Clinical outcomes were summarized according to laxative use and its various subgroups [Table 2]. Age-adjusted incidence rates of falls, total and hip fractures per 1000 person-years were calculated according to duration of laxative use by direct-adjustment based on the 5-year age distribution of the total CTs+OS sample (n=161,808) [Additional file 1: Table S1]. Hazard ratios (HRs) for the risk of falls (≥2) and total or hip fractures associated with laxative use at baseline as compared with no laxative use were obtained by adjusted Cox proportional hazards analyses using time from enrollment to the first occurrence of the event as 'time' in the models [Table 3]. Two models were formed for each outcome to examine the effect of potential confounding [15,16,57,58]. Model 1 was stratified by 10-year age intervals and OS vs. CT cohorts, and adjusted for linear age, race/ethnicity, and randomization assignment for WHI-CTs. Model 2A additionally adjusted for BMI, smoking, physical activity, self-reported health, treated diabetes mellitus, history of fracture at age ≥55 years, corticosteroid use, physical function score, number of chronic medical conditions (including treated diabetes mellitus, stroke, any cancer, CVD, arthritis, hypertension, and emphysema), number of psychoactive medications (including anxiolytics, hypnotics, antidepressants, antipsychotics, and antiepileptic agents), use of HT and bisphosphonates. Model 2B also included history of falls (≥2). Similarly, we constructed adjusted hazards ratio of falls and bone outcomes using time-dependent laxative use from all medication inventories collected during follow-up time [Additional file 1: Table S2]. Data on laxative use were updated at year 3 in the OS, and at years 1, 3, 6 and 9 in the CT. Follow-up time was censored three years after last medication collection. Multivariate linear regression methods were constructed to assess the association of baseline BMD for total hip, total spine and total body levels with any laxative use, as well as 3-year changes in BMD [Table 4]. For evaluating the relation of the duration of laxative intake to the incidence of falls and fractures, we used proportional hazards models to examine the association of different duration of laxative use (<1 year, 1–3 years, or >3years) with the outcomes of interest [Table 5]. We also conducted selected subgroup analyses for associations of laxative use on the outcomes by age group, current hormone replacement therapy use, current psychoactive therapy, BMI and history of fracture [Additional file 1: Table S3]. Finally, to further explore laxative intake under randomized trial conditions we conducted subgroup analyses relating any laxative use to incidence of fracture and falls in the WHI Hormone Therapy Trial [Additional file 1: Table S4]. All analyses were conducted using SAS statistical software (version 9.2; SAS Institute Inc, Cary, North Carolina).

**Table 1:** Baseline differences between users and non-users of laxatives

	**Laxative user (N=8907)**	**Nonuser of laxatives (N=151,497)**	**P-value**
Age			
< 60	-	51188 (33.8%)	< 0.0001
>= 60	6745 (75.7%)	100309 (66.2%)	
Race/ethnicity			
White	7859 (88.2%)	124468 (82.2%)	< 0.0001
Black	630 (7.1%)	13899 (9.2%)	
Other	418 (4.7%)	13130 (8.7%)	
BMI (kg/m2)			
<25	3334 (37.7%)	52350 (34.9%)	< 0.0001
25 - <30	3079 (34.8%)	52168 (34.7%)	
>=30	2433 (27.5%)	45624 (30.4%)	
Smoking			
Never	4353 (49.5%)	76367 (51.1%)	< 0.0001
Past	3995 (45.4%)	62554 (41.8%)	
Current	451 (5.1%)	10581 (7.1%)	
> 7 Alcoholic drinks/week			
Nondrinker	3885 (43.7%)	63883 (42.3%)	0.0252
No	3972 (44.7%)	69542 (46.1%)	
Yes	1026 (11.6%)	17568 (11.6%)	
Coffee intake			
0 cups a day	3977 (45.1%)	61373 (40.9%)	< 0.0001
<= 1 cup a day	1428 (16.2%)	23379 (15.6%)	
1-3 cups a day	2567 (29.1%)	47787 (31.9%)	
> 4 cups a day	848 (9.6%)	17359 (11.6%)	
Diabetes			
No	8482 (95.3%)	144679 (95.6%)	0.2068
Yes	418 (4.7%)	6680 (4.4%)	
Physical activity (METs) h/wk			
0, Inactive	1259 (14.6%)	22956 (15.9%)	< 0.0001
< 5	1712 (19.8%)	30445 (21.1%)	
5-12	2083 (24.1%)	34366 (23.8%)	
>=12	3584 (41.5%)	56535 (39.2%)	
Last medical visit within one year			
No	997 (11.5%)	26958 (18.4%)	< 0.0001
Yes	7683 (88.5%)	119360 (81.6%)	
Fair or poor self-reported health			
No	7738 (87.4%)	137171 (91.1%)	< 0.0001
Yes	1119 (12.6%)	13339 (8.9%)	
Osteoporosis			
No	7881 (89.4%)	138322 (92.7%)	< 0.0001
Yes	932 (10.6%)	10972 (7.3%)	
Physical functioning > 90			
No	6190 (70.7%)	92769 (62.4%)	< 0.0001
Yes	2567 (29.3%)	55808 (37.6%)	
Number of chronic conditions^a^			
0	1771 (19.9%)	43185 (28.5%)	< 0.0001
1	3016 (33.9%)	53641 (35.4%)	
2	2393 (26.9%)	34652 (22.9%)	
3	1226 (13.8%)	14609 (9.6%)	
>= 4	501 (5.6%)	5410 (3.6%)	
History of falls			
No	7702 (86.5%)	133542 (88.1%)	< 0.0001
Yes	1205 (13.5%)	17955 (11.9%)	
Fracture on or after age 55			
No	5818 (81.9%)	96946 (84.3%)	< 0.0001
Yes	1290 (18.1%)	17991 (15.7%)	
Hormone therapy use			
Never used	3233 (36.3%)	66953 (44.2%)	< 0.0001
Past user	1692 (19.0%)	24005 (15.9%)	
Current user	3977 (44.7%)	60405 (39.9%)	
Bisphosphonates use			
No	8679 (97.4%)	148671 (98.1%)	< 0.0001
Yes	228 (2.6%)	2824 (1.9%)	
Corticosteroid use			
No	8813 (98.9%)	150222 (99.2%)	0.0319
Yes	94 (1.1%)	1273 (0.8%)	
Calcitonin use			
No	8861 (99.5%)	151077 (99.7%)	< 0.0001
Yes	46 (0.5%)	418 (0.3%)	
Psychoactive drug use			
No	7123 (80.0%)	136454 (90.1%)	< 0.0001
Yes	1748 (20.0%)	15043 (9.9%)	
Total baseline calcium intake (mg/d)			
<400	458 (5.3%)	11502 (7.8%)	< 0.0001
400 - <800	1850 (21.4%)	38580 (26.3%)	
800 - <1200	2145 (24.8%)	36436 (24.8%)	
>= 1200	4202 (48.5%)	60392 (41.1%)	
Calcium supplement use			
No	2974 (33.4%)	68257 (45.1%)	< 0.0001
Yes	5933 (66.6%)	83238 (54.9%)	
Total baseline Vitamin D intake (IU/d)			
<200	2557 (29.5%)	56042 (38.1%)	< 0.0001
200 - <400	1457 (16.8%)	27342 (18.6%)	
400 - <600	2440 (28.2%)	35784 (24.4%)	
>= 600	2201 (25.4%)	27742 (18.9%)	
Vitamin D supplement use			
No	3647 (40.9%)	80042 (52.8%)	< 0.0001
Yes	5260 (59.1%)	71453 (47.2%)	
Baseline total body bone density^b^	1.017	1.014	0.5236
Baseline total body bone density adjusted^c^	1.027	1.012	0.0017
Baseline lumbar spine bone density^b^	0.990	0.979	0.1551
Baseline lumbar spine bone density adjusted^c^	0.998	0.978	0.0065
Baseline total hip bone density^b^	0.837	0.853	0.0123
Baseline total hip bone density adjusted^c^	0.850	0.850	0.9395
Hormone therapy trial			
No	7694 (86.4%)	125585 (82.9%)	< 0.0001
Yes	1213 (13.6%)	25912 (17.1%)	
Dietary modification trial			
No	6652 (74.7%)	105272 (69.5%)	< 0.0001
Yes	2255 (25.3%)	46225 (30.5%)	
Calcium Vitamin D trial			
No	7435 (83.5%)	116920 (77.2%)	< 0.0001
Yes	1472 (16.5%)	34577 (22.8%)	
Observational study			
No	3168 (35.6%)	64447 (42.5%)	< 0.0001
Yes	5739 (64.4%)	87050 (57.5%)	

^a^ Includes treated diabetes mellitus, stroke, any cancer, CVD, arthritis, hypertension, 2 or more falls, emphysema.

^b^ From subset.

^c^ Adjusted for age, ethnicity, and BMI.

**Table 2:** Clinical outcomes according to laxative use and drug class

**Clinical outcome**	**No Laxative use (N=151497)**	**Laxative use (N=8907)**	**Oral stimulant containing laxative (N=1603)**	**Fiber containing laxative (N=5372)**	**Osmotic containing laxative (N=281)**	**Stool softener containing laxative (N=2525)**	**Lubricant containing laxative (N=33)**
**Falls (> 2)**	50175 (33.9%)	3484 (40.0%)	588 (37.7%)	2040 (38.8%)	106 (39.3%)	1067 (43.3%)	15 (46.9%)
p-yrs	959,729	53,592					
Follow-up yrs (SD) / person	6.4 (2.7)	6.1 (2.7)					
**Hip fracture**	1706 (1.1%)	142 (1.6%)	33 (2.1%)	73 (1.4%)	4 (1.4%)	52 (2.1%)	0 (0.0%)
p-yrs	1,171,740	67,909					
Follow-up yrs (SD) / person	7.8 (1.6)	7.6 (1.7)					
**Total fracture**	21642 (14.3%)	1476 (16.6%)	255 (15.9%)	852 (15.9%)	48 (17.1%)	449 (17.8%)	6 (18.2%)
p-yrs	1,053,650	61,029					
Follow-up yrs (SD) / person	7.2 (2.2)	7.1 (2.2)					

**Table 3:** Adjusted hazards ratio of fractures and falls by laxative use at baseline

		Model 1 HR (95% CI)*	Model 1 p-value*	Model 2A HR (95% CI)**	Model 2A p-value**	Model 2B HR (95% CI)**	Model 2B p-value**
Falls (≥ 2)	Any laxative use	1.21 (1.17, 1.25)	<.0001	1.06 (1.03, 1.10)	0.0006	1.06 (1.03, 1.10)	0.0008
	Oral stimulant containing laxative	1.15 (1.06, 1.25)	0.0009	0.98 (0.90, 1.07)	0.6162	0.97 (0.89, 1.05)	0.4450
	Fiber containing laxative	1.15 (1.10, 1.20)	<.0001	1.06 (1.01, 1.11)	0.0147	1.05 (1.01, 1.10)	0.0272
	Osmotic containing laxative	1.20 (0.99, 1.46)	0.0577	1.10 (0.90, 1.34)	0.3493	1.10 (0.90, 1.34)	0.3510
	Stool softener containing laxative	1.35 (1.27, 1.44)	<.0001	1.08 (1.02, 1.15)	0.0145	1.10 (1.03, 1.17)	0.0036
	Lubricant containing laxative	1.51 (0.91, 2.50)	0.1113	1.10 (0.63, 1.94)	0.7390	1.06 (0.60, 1.87)	0.8377
Hip fracture	Any laxative use	1.11 (0.94, 1.32)	0.2200	1.02 (0.85, 1.22)	0.8509	1.02 (0.85, 1.22)	0.8426
	Oral stimulant containing laxative	1.62 (1.15, 2.28)	0.0062	1.31 (0.90, 1.91)	0.1556	1.32 (0.91, 1.93)	0.1444
	Fiber containing laxative	0.92 (0.73, 1.16)	0.4907	0.86 (0.67, 1.11)	0.2514	0.87 (0.67, 1.11)	0.2552
	Osmotic containing laxative	1.06 (0.40, 2.82)	0.9118	0.98 (0.37, 2.61)	0.9625	1.00 (0.38, 2.68)	0.9942
	Stool softener containing laxative	1.46 (1.11, 1.93)	0.0070	1.26 (0.94, 1.69)	0.1203	1.26 (0.94, 1.69)	0.1273
	Lubricant containing laxative	NA***					
Total fracture	Any laxative use	1.07 (1.01, 1.13)	0.0137	1.01 (0.96, 1.07)	0.6237	1.02 (0.96, 1.07)	0.5511
	Oral stimulant containing laxative	1.13 (0.99, 1.27)	0.0606	1.04 (0.92, 1.18)	0.5391	1.04 (0.91, 1.18)	0.5725
	Fiber containing laxative	0.99 (0.92, 1.06)	0.6831	0.97 (0.90, 1.04)	0.4089	0.97 (0.90, 1.04)	0.4004
	Osmotic containing laxative	1.15 (0.87, 1.53)	0.3263	1.10 (0.82, 1.47)	0.5285	1.12 (0.84, 1.49)	0.4517
	Stool softener containing laxative	1.20 (1.09, 1.31)	0.0002	1.05 (0.95, 1.16)	0.3285	1.06 (0.96, 1.17)	0.2331
	Lubricant containing laxative	1.29 (0.58, 2.88)	0.5267	1.29 (0.58, 2.87)	0.5322	1.26 (0.57, 2.81)	0.5665

* Adjusted for age, ethnicity, and WHI clinical trial indicators.

** Adjusted for age, ethnicity, BMI, WHI clinical trial indicators, smoking status, physical activity, self-reported health, treated diabetes, history of fracture after age 55, corticosteroid use, physical function score, number of chronic medical conditions (including treated diabetes mellitus, stroke, any cancer, CVD, arthritis, hypertension, and emphysema), number of psychoactive drugs (including antipsychotic, antiepileptic, anxiolytic, hypnotic and antidepressant drug use), use of HRT, and bisphosphonate use, and in Model 2B history of falls (≥2).

*** No hip fractures occurred in this group.

**Table 4:** Three-year changes in mean bone mineral density (BMD) according to laxative use

	**Laxative Use**		**No Laxative Use**				
	**N**	Mean (SE) *	**N**	Mean (SE) *	P-value *	P-value **	P-value ***
Total hip							
Baseline	509	0.842 (0.006)	10791	0.847 (0.001)	0.4170	0.8376	0.8624
Year 3	424	0.847 (0.006)	8916	0.854 (0.001)	0.2767	0.3282	0.3409
Absolute change	422	0.004 (0.002)	8810	0.006 (0.000)	0.3866	0.3734	0.3714
Percent change	422	0.596 (0.201)	8810	0.730 (0.047)	0.5108	0.4856	0.4791
Total spine							
Baseline	487	0.991 (0.007)	10456	0.975 (0.002)	0.0339	0.1307	0.1325
Year 3	401	1.010 (0.009)	8653	0.995 (0.002)	0.0836	0.4034	0.3974
Absolute change	400	0.017 (0.002)	8579	0.021 (0.001)	0.2075	0.1396	0.1573
Percent change	400	1.840 (0.257)	8579	2.198 (0.060)	0.1711	0.1324	0.1505
Total body							
Baseline	512	1.023 (0.004)	10794	1.011 (0.001)	0.0090	0.0209	0.0234
Year 3	424	1.033 (0.005)	8891	1.023 (0.001)	0.0692	0.1754	0.1854
Absolute change	423	0.012 (0.002)	8796	0.011 (0.000)	0.6686	0.9132	0.8580
Percent change	423	1.236 (0.181)	8796	1.150 (0.043)	0.6422	0.8545	0.7946

* Adjusted for age, ethnicity, and WHI clinical trial indicators.

** Adjusted for age, ethnicity, BMI, WHI clinical trial indicators, smoking status, physical activity, self-reported health, treated diabetes, history of fracture after age 55, corticosteroid use, physical function score, number of chronic medical conditions (including treated diabetes mellitus, stroke, any cancer, CVD, arthritis, hypertension, and emphysema), number of psychoactive drugs (including antipsychotic, antiepileptic, anxiolytic, hypnotic and antidepressant drug use), use of HRT, and bisphosphonate use.

*** Adjusted as in (**) with the addition of history of falls (≥ 2).

**Table 5:** Adjusted hazards ratio of fractures and falls by any laxative use duration

**Any laxative use**	**N**	HR (95% CI)**		
		**Falls (>2)**	**Hip fracture**	**Total fracture**
Nonuser	151,497	1	1	1
< 1 year	1924	1.11 (1.04, 1.74)	1.17 (0.89, 1.55)	1.06 (0.97, 1.16)
1-3 years	3091	1.07 (0.99, 1.15)	1.02 (0.69, 1.50)	1.03 (0.92, 1.15)
> 3 years	3892	1.00 (0.95, 1.06)	0.90 (0.68, 1.19)	0.97 (0.89, 1.05)
p-value for linear trend		0.0458	0.9553	0.9996

** Adjusted for age, ethnicity, BMI, WHI clinical trial indicators, smoking status, physical activity, self-reported health, treated diabetes, history of fracture after age 55, corticosteroid use, physical function score, number of chronic medical conditions (including treated diabetes mellitus, stroke, any cancer, CVD, arthritis, hypertension, 2 or more falls and emphysema), number of psychoactive drugs (including antipsychotic, antiepileptic, anxiolytic, hypnotic and antidepressant drug use), use of HRT, and bisphosphonate use.

## Results

Among the 161,808 women in the WHI OS and CTs, 160,404 had no prior hip fracture at baseline and were included in this study; 8907 (5.6%) of study participants were currently using laxatives at baseline [Table 1]. Of those, 1603 persons used an oral stimulant containing laxative, 5372 a fiber containing laxative, 281 an osmotic containing laxative, 2525 a stool softener containing laxative and 33 a lubricant containing laxative [Table 2]. Nearly 4000 women (43.7%) had used laxative medication for more than 3 years, 3091 (34.7%) for 1 to 3 years, and 1924 (21.6%) for less than 1 year [Table 5]. Those using laxatives at baseline were of older age, had lower BMI, more likely to have a lower physical functioning or a history of fractures, more likely to report low or fair health or osteoporosis, and to be included in the observational study (WHI-OS) [Table 1]. Concurrent use of psychoactive medications and hormone replacement therapy was more common among laxative users. Calcium and Vitamin D supplementation as well as bisphosphonates and calcitonin were more often used by laxative users. After adjustment for age, ethnicity and BMI laxative users tended to have a higher lumbar spine and total BMD. Other baseline characteristics were similar.

During 6.4 follow-up years/person in the laxative non-user group (n=151,497) as compared to 6.1 follow-up years/person in the user group (n=8907), 50,175 falls were reported in the non-user and 3484 falls in the user-group [Table 2]. The age-adjusted rates of falls were 52.84/1000 person-years for non-users and 63.35/1000 person-years for laxative users. Similarly, the rates of hip fractures were 1.54/1000 and 1.75/1000 and the rates of total fractures were 20.89/1000 and 23.07/1000 for laxative non-users and users [Additional file 1: Table S1].

In minimally adjusted models a significant association was found between ‘any laxative use’ and falls (HR, 1.21; 95% CI, 1.17-1.25), and total fractures (HR, 1.07; 95% CI, 1.01-1.13) at baseline, while the association with hip fractures was not statistically significant (HR, 1.11; 95% CI, 0.94-1.32) [Table 3].When stratifying these associations by the various laxative subgroups, we noted ‘oral stimulant’, ‘fiber’ and ‘stool softener containing laxatives’ to be significantly associated with the risk of falls. After full adjustment ‘any laxative use’ (Model 2A: HR, 1.06; 95% CI, 1.03-1.10), ‘fiber containing laxatives’ (Model 2A: HR, 1.06; 95% CI, 1.01-1.11) and ‘stool softener containing laxatives’ (Model 2A: HR, 1.08; 95% CI, 1.02-1.15) remained statistically significantly associated with falls. These results did only marginally change after additional adjustment for history of falls. No significant relationship between laxative use and the risk for fractures was found after fully accounting for confounding factors. To investigate the effects of laxative use over time, we additionally constructed time-dependent models [Additional file 1: Table S2]. There we continued to find a significant association between the risk for falls and ‘any laxative use’ (HR, 1.06; 95% CI, 1.02-1.10) and ‘stool softener containing laxatives’ (HR 1.13; 95% CI, 1.06-1.21). In contrast to baseline analyses, ‘oral stimulant containing laxatives’ (HR, 2.04; 95% CI, 1.36-3.07) and ‘stool softener containing laxatives’ (HR, 1.50; 95% CI, 1.09-2.05) remained significantly associated with the risk for hip fractures even after full adjustment. No relationship between the risk for total fractures and any laxative subgroup except for ‘stool softener containing laxatives’ (HR, 1.12; 95% CI, 1.00-1.25) could be established in time-dependent analyses.

At baseline, women using laxatives had similar BMD at the hip level (adjusted mean, 0.85 vs. 0.84 g/cm^2^) compared with nonusers. Total body BMD and total spine BMD differed significantly in users compared with nonusers (adjusted mean, 1.02 vs. 1.01 g/cm^2^ and 0.99 vs. 0.98 g/cm^2^) [Table 4]. Examining the 3-year BMD change from baseline, we found small statistically non-significant increases in BMD in all areas of interest, which may stem from the fact that a high proportion of women in both groups were part of the active treatment arms in the WHI-CT involving calcium and vitamin D supplementation and/or hormone trial (HT).

Any laxative use tended to be associated with an increased hazard for falls in stratified analysis by exposure duration while no clear trend could be observed for the risk for hip fractures or total fractures [Table 5]. Because laxative use may have different effects on various subpopulations, we additional undertook several subgroup analyses [28,59,60]. Laxative use was associated with an increased hazard for total fractures by age in women older than 65 years (HR, 1.06; 95% CI, 0.99-1.14, interaction p-value = 0.05), although no significant interaction by age was found for falls and hip fractures. Psychoactive therapy was associated with a lower risk for total fractures (HR, 0.91; 95% CI, 0.81-1.03, interaction p-value = 0.05), whereas no significant influence on falls or hip fractures was noted [Additional file 1: Table S3]. Finally, we found no significant difference in the prevalence of laxative intake between women receiving hormone therapy or placebo under WHI Hormone Therapy Trial settings [Additional file 1: Table S4]. Interaction tests for women stratified by trial randomization did not result in significant differences in risk of falls and fractures between laxative users and non-users.

## Discussion

In this large prospective study of postmenopausal women with no prior hip fracture, we found a statistically significant association of laxative use with the occurrence of falls (≥2). Laxative use was not associated with an increased risk for total fractures and hip fractures after fully adjustment for confounding factors. Among laxative users at baseline, the HR for falls was 1.06, for hip fractures 1.02 and for total fractures 1.01. The CIs indicate that the true HR for falls may be as low as 1.03 or as high as 1.10, for hip fractures 0.85 and 1.22 and for total fractures 0.96 and 1.07 respectively. Using time-dependent analyses laxative use remained statistically significantly associated with an increased risk for falls. These results were most likely driven by a higher number of women starting to take laxatives during follow-up; thus we achieved a higher power to detect differences between users and non-users. Nonetheless, we cannot rule out residual confounding by indication as having partly influenced our results. Longer duration of laxative use did not show a statistically significant association with the risk for hip fractures or total fractures, while a positive relationship for falls could be observed.

Several intrinsic and extrinsic effects of laxatives have been associated with their use providing potential mechanisms for an altered risk for falls and bone outcomes [2,5,9,25,28,61]. Extrinsically, laxatives can increase the urgency to void with resulting higher risk of falling. Intrinsically, laxatives can lead to diarrhoea with associated electrolyte and fluid imbalances. Potassium is the main electrolyte being lost followed by reductions in sodium and chloride. Generalized muscle weakness, lassitude and cardiac arrhythmias may be a direct consequence of electrolyte disturbances resulting in an increased risk for falls and bone outcomes [8-14,32,35-38]. Most recent reports have linked electrolyte imbalances such as mild hyponatremia to osteoporosis and fracture risk [9-11,30,61-63]. Additionally, laxatives have also been described to irritate bowel functioning and bowel disturbances in the colon and small bowl [2,3,28]. Fat-soluble vitamin malabsorption secondary to these types of laxative induced side effects of small intestine damage may further predispose for falls, fractures and BMD loss [13,14,32-35,37]. Indeed, prior epidemiological studies have shown that laxative use is associated with an increased risk for falls [2,20-28]. One of the first was the St. Louis OASIS study in which laxative use was found to be an important risk factor for multiple falls in elderly people living in the community (OR, 2.14; 95% CI, 1.02-4.49) [59]. Other cross-sectionally designed studies confirmed this positive association [22,23,27]. Observational studies examining medications and chronic diseases with one year follow-up period reported risks for falling between OR 1.09 (95% CI, 0.86-1.38) and 2.51 (95% CI, 1.50-4.22) with laxative use [22,23,25,26,29]. A recent meta-analysis summarizing the risks for falls related to laxatives elderly individuals resulted in an OR 2.03 (95% CI, 1.52-2.72) [28]. Nevertheless, external validity of many included studies may be limited as the mean age of the study population was above 80 years old [20,22,27,29] with a small number of participants, events and short follow-up [20,22]. Last, data on fracture risk or change in BMD were not assessed even though a history of falls is an independent risk factor for fractures [39].

In contrast to previously published findings [28,59], our results indicate a modest association between laxative use and the occurrence of falls, whereas we found no relationship between laxative use and fractures. Furthermore, although baseline total body bone density and total lumbar spine density varied between laxative users or non-users, three-year changes in BMD did not show any significant differences between both groups for all BMD areas. In particular, we did not observe differences in total hip bone density between laxative users or not users. These findings are surprising given the different baseline characteristics of laxative users and non-users suggesting that laxative users have a higher number of chronic conditions, lower physical functioning and are of older age, factors known to influence the risk for falls, fractures and changes in BMD [26,64,65]. It may be explained by various levels of calcium and vitamin D intake between the two groups. Nevertheless, given our hypothesis that laxative intake may potentially cause electrolyte imbalances, vitamin D malabsorption or even small intestine mucosal damage, we expected a strong relationship between falls and laxative use and an increased risk for fractures and BMD reductions. To further investigate the association of laxative use with the incidence of falls and fractures when used concomitantly with certain medications (psychoactive drugs, hormone therapy), in certain age groups, with different BMI levels or history of fracture, we conducted a subgroup analysis. We found a slightly higher risk for falls in laxative users being of older age (>65 y), while current psychoactive therapy, BMI, history of fracture had no negative effect. Moreover, we noted a higher risk for falls in laxative users under current hormone treatment in the total study population (WHI OS and CTs) which we and others did not continue to observe in a hormone trial setting [66]. On the other hand, older women using laxatives showed a significant risk for total fractures despite similar BMD levels. Finally, concomitant use of psychoactive agents did not increase the risk for hip and total fractures in our study population.

The strengths of our analysis include the sample size of our study population, the large number of complete follow-up and the structured availability of several confounding factors. Nonetheless, our study also exhibits several limitations. Among those, first the relative low prevalence of long time laxative use and the missing data on dosing may limit the validity of this study. Also, since many commercially available laxatives are mixture products consisting of two or more pharmacologically active substances, our analyses on the various categories of laxatives are limited due to missing information on the exact composition of these products. Second, laxative intake as well as incident falls and fractures were ascertained via self-report. Hence, we cannot exclude information bias. However, since laxative intake is likely to be assumed to be underreported, the actual effects may be underestimated. Third, even though a wide range of confounding factors was considered in the analysis, residual confounding cannot be excluded. Our results may be partly confounded by indication for which we controlled by measures of restriction, stratification and extensive multivariate adjustment. Additionally, even though this study was designed prospectively, reverse causation may have influenced our results. Last, as our study population consisted of postmenopausal women, generalizability may be limited and further research in other study populations is warranted.

## Conclusions

Laxative users are characterized by older age, lower physical functioning, more comorbidities and higher concurrent use of psychoactive medications and hormone replacement therapy. Our findings support an association between any laxative use and increased risk for falls but not with total fracture risk or hip fracture risk. A relationship of laxative use with changes in BMD over time was not demonstrated, but calcium and vitamin D were more often supplemented by laxative users. Bisphosphonate and calcitonin use was more likely in this group as well. Thus, questions remain regarding the influence on bone health and potential risks associated with long-term laxative use. Given the low cost, widespread use and over-the-counter availability it appears prudent to periodically reevaluate laxative use. Maintaining physical functioning, and providing adequate treatment of comorbidities that predispose individuals for falls should be considered as first measures to avoid potential negative consequences associated with laxative use.

## Competing interests

Dr. Carnahan's work was supported by the University of Iowa Institute for Clinical and Translational Science, and an Agency for Healthcare Research and Quality Centers for Education and Research on Therapeutics cooperative agreement #5 U18 HS016094 (the Iowa Older Adults CERT). In the past 5 years he received research funding from Boehringer Ingelheim, Forest Laboratories, Wyeth, and Ortho-McNeil. None of the other authors reported disclosures.

## Authors’ contributions

MP and BH had full access to all of the data in the study and take responsibility for the integrity of the data and the accuracy of the data analysis. Study concept and design: BH, SWW Acquisition of data: BP, MP, SWW Analysis and interpretation of data: MP, BH, WvB, JWB, JWW, RMC, RBW Drafting of the manuscript: BH, JWB, JWW, JO, RBW Critical revision of the manuscript for important intellectual content: JWB, RCM, JO, RCM, RBW, SWW, WvB Statistical analysis: MP Obtained funding: SWW Administrative, technical, or material support: SWW Study supervision: SWW. All authors read and approved the final manuscript.

## Pre-publication history

The pre-publication history for this paper can be accessed here:

http://www.biomedcentral.com/1471-2318/13/38/prepub

## Supplementary Material

Additional file 1: Table S1 Age adjusted rates for any laxative use at baseline. **Table S2** Adjusted hazards ratio of fractures and falls by laxative use using time-dependent laxative use from all medication inventories collected during follow-up. **Table S3** Adjusted hazard ratios relating any laxative use to incidence of fracture and falls - subgroup analyses and interaction tests. **Table S4** Adjusted hazard ratios relating any laxative use to incidence of fracture and falls in the WHI Hormone Therapy trial – subgroup analyses and interaction tests. Click here for file
