# Supplementary material for: Laxative use and incident falls, fractures and change in bone mineral density in postmenopausal women: results from the Women’s Health Initiative
Source: BMC Geriatr. 2013 May 1;13:38. doi: 10.1186/1471-2318-13-38 (PMC3645973; doi:10.1186/1471-2318-13-38)
Supplement: Additional file 1: Table S1 — Age adjusted rates for any laxative use at baseline. Table S2 Adjusted hazards ratio of fractures and falls by laxative use using time-dependent laxative use from all medication inventories collected during follow-up. Table S3 Adjusted hazard ratios relating any laxative use to incidence of fracture and falls - subgroup analyses and interaction tests. Table S4 Adjusted hazard ratios relating any laxative use to incidence of fracture and falls in the WHI Hormone Therapy trial – subgroup analyses and interaction tests. [file 1471-2318-13-38-S1.doc]

**Title:** Laxative use and incident falls, fractures and change in bone mineral density in postmenopausal women: Results from the Women’s Health Initiative.

**Authors:**

B Haring,^1^ M Pettinger,^2^ J W Bea,^3^ J Wactawski-Wende,^4^ R M Carnahan,^5^J K Ockene,^6^ M Wyler von Ballmoos,^7^ R B Wallace,^5^ S Wassertheil-Smoller,^8^

**Author Affiliations:**

^1^ Comprehensive Heart Failure Center, Department of Internal Medicine I, University of Würzburg, Würzburg, Bavaria, Germany. E-mail: Haring_B@medizin.uni-wuerzburg.de

^2^ Fred Hutchinson Cancer Research Center, Seattle, Washington, USA. E-mail: mpettinger@whi.org

^3^Arizona Cancer Center, University of Arizona, Tucson, Arizona, USA. E-mail: jbea@azcc.arizona.edu

^4^ Department of Social and Preventive Medicine, University of Buffalo, SUNY, Buffalo, New York, USA. E-mail: jww@buffalo.edu

^5^Department of Epidemiology, University of Iowa College of Public Health, Iowa City, Iowa, USA. E-mail: ryan-carnahan@uiowa.edu

^6^Division of Preventive and Behavioral Medicine, University of Massachusetts Medical School, Worcester, Massachusetts, USA. E-mail: Judith.Ockene@umassmed.edu

^7^ Department of Cardiothoracic Surgery, Medical College of Wisconsin, Milwaukee, Wisconsin, USA. E-mail: [mcwvb@post.harvard.edu](mailto:mcwvb@post.harvard.edu)

^8^ Department of Epidemiology, University of Iowa College of Public Health, Iowa City, Iowa, USA. E-mail: robert-wallace@uiowa.edu

^9^ Department of Epidemiology and Population Health, Albert Einstein College of Medicine, Bronx, New York, USA. E-mail: Sylvia.Smoller@einstein.yu.edu

**Supplements:**

Table S1: Age adjusted rates for any laxative use at baseline

Table S2: Adjusted hazards ratio of fractures and falls by laxative use using time-dependent laxative use from all medication inventories collected during follow-up

Table S3: Adjusted hazard ratios relating any laxative use to incidence of fracture and falls - subgroup analyses and interaction tests

Table S4: Adjusted hazard ratios relating any laxative use to incidence of fracture and falls in the WHI Hormone Therapy trial – subgroup analyses and interaction tests

**Supplements**

# **Table S1: Age adjusted* rates for any laxative use at baseline**

|  | Any Laxative Use at Baseline | | | | |
| --- | --- | --- | --- | --- | --- |
|  | Nonuser  (n=151,497) | Any use  (n=8907) | Duration of use | | |
|  |  |  | < 1 yr of use  (n=1924) | 1-3 yrs of use  (n=3091) | 3 yrs of use  (n=3892) |
| Falls (>2) |  |  |  |  |  |
| N | 50175 | 3484 | 745 | 1251 | 1488 |
| Person years of follow-up | 959,729 | 53,592 | 11,550 | 18,362 | 23,680 |
| Age-adjusted rate/1000 person-years | 52.84 | 63.35 | 63.40 | 66.27 | 61.22 |
| Hip Fracture |  |  |  |  |  |
| N | 1706 | 142 | 29 | 57 | 56 |
| Person years of follow-up | 1,171,740 | 67,909 | 14,679 | 23,445 | 29,785 |
| Age-adjusted rate/1000 person-years | 1.54 | 1.75 | 1.70 | 2.10 | 1.52 |
| Total Fracture |  |  |  |  |  |
| N | 21642 | 1476 | 325 | 526 | 625 |
| Person years of follow-up | 1,053,650 | 61,029 | 13,247 | 21,092 | 26,690 |
| Age-adjusted rate/1000 person-years | 20.89 | 23.07 | 23.84 | 23.60 | 22.24 |

* Direct-adjustment based on the 5-year age distribution of the total CT+OS sample (n=161,808)

**Table S2: Adjusted hazards ratio of fractures and falls by laxative use using time-dependent laxative use from all medication inventories collected during follow-up***

|  | | Model 1 HR (95% CI)* | Model 1 p-value** | Model 2 HR (95% CI)** | Model 2 p-value*** |
| --- | --- | --- | --- | --- | --- |
| Falls (≥ 2) | Any laxative use | 1.21 (1.17, 1.26) | <.0001 | 1.06 (1.02, 1.10) | 0.0042 |
|  | Oral stimulant containing laxative | 1.13 (1.02, 1.24) | 0.0168 | 0.98 (0.89, 1.09) | 0.6937 |
|  | Fiber containing laxative | 1.11 (1.06, 1.17) | <.0001 | 1.01 (0.96, 1.06) | 0.8302 |
|  | Osmotic containing laxative | 1.18 (0.95, 1.47) | 0.1306 | 1.07 (0.86, 1.34) | 0.5375 |
|  | Stool softener containing laxative | 1.40 (1.31, 1.49) | <.0001 | 1.13 (1.06, 1.21) | 0.0003 |
|  | Lubricant containing laxative | 1.62 (0.92, 2.85) | 0.0919 | 1.30 (0.72, 2.36) | 0.3784 |
|  |  |  |  |  |  |
| Hip fracture | Any laxative use | 1.33 (1.11, 1.60) | 0.0023 | 1.21 (0.99, 1.47) | 0.0624 |
|  | Oral stimulant containing laxative | 2.31 (1.56, 3.40) | <.0001 | 2.04 (1.36, 3.07) | 0.0006 |
|  | Fiber containing laxative | 0.98 (0.74, 1.29) | 0.8591 | 0.94 (0.70, 1.27) | 0.6985 |
|  | Osmotic containing laxative | 1.66 (0.69, 3.99) | 0.2603 | 1.60 (0.67, 3.87) | 0.2926 |
|  | Stool softener containing laxative | 1.84 (1.38, 2.46) | <.0001 | 1.50 (1.09, 2.05) | 0.0121 |
|  | Lubricant containing laxative | NA**** |  |  |  |
|  |  |  |  |  |  |
| Total fracture | Any laxative use | 1.06 (1.00, 1.13) | 0.0495 | 1.01 (0.95, 1.07) | 0.7932 |
|  | Oral stimulant containing laxative | 1.15 (0.98, 1.34) | 0.0790 | 1.03 (0.88, 1.21) | 0.7137 |
|  | Fiber containing laxative | 0.94 (0.87, 1.02) | 0.1582 | 0.93 (0.85, 1.01) | 0.0668 |
|  | Osmotic containing laxative | 1.12 (0.80, 1.55) | 0.5120 | 1.14 (0.83, 1.59) | 0.4199 |
|  | Stool softener containing laxative | 1.26 (1.13, 1.39) | <.0001 | 1.12 (1.00, 1.25) | 0.0459 |
|  | Lubricant containing laxative | 1.90 (0.86, 4.24) | 0.1148 | 1.77 (0.79, 3.94) | 0.1625 |

* Laxative use was updated at year 3 in the OS, and at years 1, 3, 6 and 9 in the CT. Follow-up time was censored three years after last medication collection.

** Adjusted for age, ethnicity, BMI and WHI clinical trial indicators.

*** Adjusted for age, ethnicity, BMI, WHI clinical trial indicators, smoking status, physical activity, self-reported health, treated diabetes, history of fracture after age 55, corticosteroid use, physical function score, number of chronic medical conditions (including treated diabetes mellitus, stroke, any cancer, CVD, arthritis, hypertension, 2 or more falls and emphysema), number of psychoactive drugs (including antipsychotic, antiepileptic, anxiolytic, hypnotic and antidepressant drug use), use of HRT, and bisphosphonate use, and in Model 2B history of falls (≥2).

**** No hip fractures occurred in this group.

**Table S4. Adjusted Hazard Ratios[[1]](#footnote-2)** Relating Any Laxative use to Incidence of Fracture and Falls in the Hormone Therapy Trial – Subgroup Analyses and Interaction Tests

|  | **Falls** | | | | | | **Hip Fracture** | | | | | | **Total Fracture** | | | | | |
| --- | --- | --- | --- | --- | --- | --- | --- | --- | --- | --- | --- | --- | --- | --- | --- | --- | --- | --- |
|  | **Non User** | | **User** | | **HR (95% CI)** | **P-Value[[2]](#footnote-3)** | **Non User** | | **User** | | **HR (95% CI)** | **P-Value^2^** | **Non User** | | **User** | | **HR (95% CI)** | **P-Value^2^** |
|  | **N** | **Ann %** | **N** | **Ann %** |  |  | **N** | **Ann %** | **N** | **Ann %** |  |  | **N** | **Ann %** | **N** | **Ann %** |  |  |
| **Hormone Therapy** | 4579 | 5.54 | 255 | 7.31 | 1.04 (0.91, 1.19) | 0.5114 | 147 | 0.14 | 9 | 0.20 | 0.90 (0.46, 1.78) | 0.3450 | 1579 | 1.62 | 84 | 1.97 | 1.04 (0.82, 1.31) | 0.7223 |
| **Placebo** | 4635 | 5.88 | 281 | 7.82 | 1.11 (0.97, 1.26) |  | 189 | 0.19 | 21 | 0.44 | 1.35 (0.81, 2.24) |  | 1953 | 2.11 | 118 | 2.69 | 1.10 (0.90, 1.34) |  |

|  | **Laxative Users (N=1,213)** | **Laxative Non-Users (N=25,912)** | **P-Value** |
| --- | --- | --- | --- |
| **Hormone Therapy** | 588 (48.5%) | 13,115 (50.6%) | 0.1453 |
| **Placebo** | 625 (51.5%) | 12,797 (49.4%) |  |

1. From a Cox proportional hazards regression model stratified by 10-year age intervals, and adjusted for linear age, race/ethnicity, BMI, hysterectomy status, history of fracture after age 55, treated diabetes, current physical activity from walking, physical function, self-reported health, smoking, number of chronic conditions and medication use (bisphosphonates, corticosteroids, number of psychoactive medications). [↑](#footnote-ref-2)
2. Tests for interaction with laxative use in a Cox proportional hazards regression model stratified and adjusted as above. [↑](#footnote-ref-3)
